# Follistatin could promote the proliferation of duck primary myoblasts by activating PI3K/Akt/mTOR signalling

**DOI:** 10.1042/BSR20140085

**Published:** 2014-10-17

**Authors:** Xinxin Li, Hehe Liu, Haohan Wang, Lingli Sun, Fang Ding, Wenqiang Sun, Chunchun Han, Jiwen Wang

**Affiliations:** *Institute of Animal Breeding and Genetic, Sichuan Agricultural University, Ya’an, Sichuan 625014, People's Republic of China; †Department of Animal Science, Henan Vocational College of Agriculture, Zhengzhou, Henan 451450, People's Republic of China

**Keywords:** duck, Follistatin, myoblasts, PI3K/Akt/mTOR signalling, proliferation, Akt, protein kinase B, BrdU, 5-bromo-2V-deoxyuridine, DAPI, 4′,6-diamidino-2-phenylindole, FST, *Follistatin*, HRP, horseradish peroxidise, IGF, insulin-like growth factor, IGF-IR, type 1 insulin-like growth factor receptor, mTOR, mammalian target of rapamycin, MSTN, myostatin, MTT, 3-(4,5-dimethylthiazol-2-yl)-2,5-diphenyltetrazolium bromide, MuRF1, muscle RING finger-1, PI3K, phosphoinositide 3-kinase, S6K, S6 kinase

## Abstract

FST (follistatin) is essential for skeletal muscle development, but the intracellular signalling networks that regulate FST-induced effects are not well defined. We sought to investigate whether FST promotes the proliferation of myoblasts through the PI3K (phosphoinositide 3-kinase)/Akt (protein kinase B)/mTOR (mammalian target of rapamycin) signalling. In the present study, we transfected the pEGFP-duFST plasmid and added PI3K and mTOR inhibitors to the medium of duck primary myoblasts. Then, we analysed the cellular phenotypic changes that occurred and analysed the expression of target genes. The results showed that FST promoted myoblast proliferation, induced the mRNA expression of PI3K, Akt, mTOR, 70-kDa ribosomal protein S6K (S6 kinase) and the protein expression of phospho-Akt (Thr^308^), mTOR, phospho-mTOR (serine 2448), phospho-S6K (Ser^417^), inhibited the mRNA expression of FoxO1, MuRF1 (muscle RING finger-1) and the protein expression of phospho-FoxO1 (Ser^256^). Moreover, we found that the overexpression of FST could alleviate the inhibitory effect of myoblast proliferation caused by the addition of LY294002, a PI3K inhibitor. Additionally, the overexpression of duck FST also relieved the inhibition of myoblast proliferation caused by the addition of rapamycin (an mTOR inhibitor) through PI3K/Akt/mTOR signalling. In light of the present results, we hypothesize that duck FST could promote myoblast proliferation, which is dependent on PI3K/Akt/mTOR signalling.

## INTRODUCTION

FST (follistatin), a single-chain monomeric glycoprotein, plays an important function in embryogenesis, muscle development and adult life [1,2]. In mammals, the overexpression of FST markedly increases muscle mass through both hyperplasia and hypertrophy of myofibres [3]. On the contrary, FST knockout mice have been shown to die immediately after birth and have a clear phenotype of muscle mass loss [4]. Additionally, it was shown that FST is important in promoting myoblast proliferation and differentiation *in vitro* studies [5]. Indeed, FST is a key functional gene that regulates muscle development, and its regulatory mechanisms in muscle development were recently studied more intensively by many researchers in the field. Some scholars have shown that FST could promote skeletal muscle hypertrophy in mice by activating IGF-1R (type 1 insulin-like growth factor receptor)/Akt (protein kinase B) signalling and inhibiting MSTN (myostatin) signalling [6,7]. However, in birds, the information about FST in muscle development has not been well studied. Previous studies in our laboratory showed that recombinant duck FST protein could promote muscle hypertrophy in the post-hatching duck by inducing satellite cell proliferation [8]. However, FST played minor roles in embryonic skeletal muscle development [9]. To date, the regulatory mechanism of FST in the skeletal muscle development of birds remains an open question.

The intracellular mechanism of skeletal muscle hypertrophy is associated with protein metabolism, including both synthesis and degradation biochemical processes [10]. Researchers have shown that PI3K (phosphoinositide 3-kinase)/Akt/mTOR (mammalian target of rapamycin) signalling plays a significant role in regulating protein metabolism. S6K (S6 kinase), a downstream effector of mTOR, is an important regulator of protein synthesis [11]. In mice, deletion of the S6K gene can lead to decreased numbers of nuclei in muscle fibres and a reduction in skeletal muscle growth [12]. FoxO1, which is located downstream of Akt, is a member of the forkhead (FKHR; FOXO) family of transcription factors [13,14]. Researchers have shown that activating FoxO1 signalling could promote protein degradation, which can further result in increased skeletal muscle atrophy [15]. The effects of environmental stresses, such as oxidative stress, genotoxic agents, growth factors and secretory proteins, can also affect muscle protein metabolism and myoblast proliferation through regulating the PI3K/Akt/mTOR signalling pathway [16–18]. IGF-1 (type 1 insulin-like growth factor), an important growth factor, was shown to activate the PI3K/Akt signal transduction pathways and mediate myotube hypertrophy [16]. Similar to IGF-1, FST is another important extracellular ligand, which has an relativeship with the IGF-IR (type 1 insulin-like growth factor receptor) pathway in promoting skeletal muscle hypertrophy in mice [6]. Therefore we speculated that the functions of FST in muscle development may be dependent on the PI3K/Akt/mTOR signalling pathway.

The purpose of this paper was to investigate whether there is a regulatory relationship between FST and PI3K/Akt/mTOR signalling that affects duck myoblast proliferation. This research may provide the new clues that are needed to clarify the regulatory mechanisms of FST in these processes. Furthermore, this research may also provide insights into the mechanism by which FST improves muscle mass and thus lead to future research on the effects of duck FST.

## MATERIALS AND METHODS

### Animals

Experimental 13-day-old Peking duck eggs (*Anas platyrhynchos domestica*) were provided by the Sichuan Agricultural University Waterfowl Breeding Experimental Farm (Ya’an, China). All of the eggs were incubated under the same conditions at a temperature of 37±0.5°C and with a humidity of 86–87%. All procedures in the present study were conducted in compliance with the requirements of the Animal Ethics Committee of Sichuan Agricultural University.

### Cell culture, treatments and transfection

According to the method previously described by Liu et al. [19], primary duck myoblasts were isolated and seeded into six-well plates at a density of 1×10^6^ cell/plate. The cells were cultured in DMEM (Dulbecco's modified Eagle's medium; Sigma-Aldrich Japan), supplemented with 10% (v/v) FBS and antibiotics (100 units/ml penicillin and 100 μg/ml streptomycin) and were maintained in an incubator with 5% (v/v) CO_2_ at 37°C. In the transfection assay, the cells were transfected with pEGFP-duFST for 24 h and were harvested for subsequent analyses. In the treatment and transfection assays, myoblasts were treated with the medium containing the inhibitors LY294002 (10 μm/ml, PI3K inhibitor) or rapamycin (20 ng/ml, mTOR inhibitor) for 24 h. Subsequently, the cells were washed twice with PBS and then transfected with pEGFP-duFST for 24 h. At the end of the incubation period, the cells were harvested and immediately frozen at −80°C for subsequent analyses.

### Myoblasts viability analysis

The viability of myoblasts was analysed using the MTT [3-(4,5-dimethylthiazol-2-yl)-2,5- diphenyltetrazolium bromide] method. In detail, 10 μl of MTT (OD_570_) (5 mg/ml, BiYunTian Biotechnology) was added to the 96-wells plates for 4 h. Then, the supernatants were removed, and 100 μl of formazan (BiYunTian Biotechnology) was added to each well. After 4 h of incubation, the absorbance value for each well was measured using a microplate reader at a wavelength of 570 nm (Thermo).

### BrdU (5-bromo-2V-deoxyuridine) assay and immunofluorescence

For the proliferation assays, myoblasts were incubated with 25 μM BrdU (10 mg/ml in PBS, Boster) for 4h at 37°C in the incubator. Immunofluorescence labelling was performed according to the method previously described by Liu et al. [19]. Briefly, each well was washed three times with PBS to remove the culture medium. Then, the myoblasts were fixed with paraformaldehyde solution (4%; v/v) and were treated with Triton X-100 solution (0.05%) in PBS for 20 min. Blocking was conducted using a blocking solution [1% (w/v) BSA in PBS] for 30 min, and the anti-BrdU antibody (antibody was diluted 1:20 with PBS; Solarbio Co.) was added to the wells and incubated overnight at 4°C. Then, the cells were washed three times with PBS and incubated with a goat anti-mouse IgG antibody (antibody was diluted 1:200 with PBS; Boster) at 37°C for 2 h. Then, the nuclei were labelled with DAPI (4′,6-diamidino-2-phenylindole; 10 μg/ml in PBS; BiYunTian Biotechnology). Finally, the myoblasts were observed using a florescence microscope (Nikon), and the photos were analysed using the Image-Pro Plus 6.0 software (Media Cybernetics).

### RNA extraction and qRT-PCR

The total RNA of the cells was isolated using Trizol (Invitrogen), following the manufacturer's instructions. For qRT-PCR analysis, the total RNA was treated with DNase I for 10 min, and the SYBR Prime Script qRT-PCR Kit (TaKaRa) was used for qRT-PCR detection. Primers were designed (Table 1) for duck FST, PI3K, Akt, mTOR, S6K, FoxO1, MuRF1, MSTN and ACVR2. GADPH (AY436595) and β-actin (EF667345) were used as the two reference genes. The reactions were carried out using the CFX96™ qRT-PCR Detection System (Bio-Rad) in 96-well plates, and the mixtures contained 1 μl of cDNA template, 12.5 μl of SYBR Premix ExTaq, 10.5 μl of sterile water and 0.5 μl of each gene-specific primer. The procedure included 30 s of a pre-denaturation reaction at 95°C, followed by 40 cycles of 95°C for 10 s and 60°C for 40 s. Each sample was repeated in triplicate. The Vandesompele method of quantification was used to calculate the expression of the target genes relative to the internal control genes.

**Table 1:** Primer sequences used for qRT-PCR F and R are forward and reverse primers, respectively.

Gene	Primer sequence (5′-3′)	Product size (bp)	*T*_m_ (°C)
FST	F: ACAACTTACCCAAGCGAGTGTG	145	58
	R: CATCTTCCTCTTCTTCCTCTGG		
PI3K	F: CTTTTACCGAGGAGGTTCTGTGG	137	60
	R: CTGAAGGTTGGTCTTTGTGGAC		
Akt	F: TCTTTGCTGGCATTGTTTGGC	152	60
	R: GCTGTCATCTTGGTCAGGAGGAGT		
mTOR	F: CTATCTGCCTCAGCTCATTCCT	121	60
	R: GTCATCCAGGTTAGCTCCAAAG		
FoxO1	F:AGGTTCACCAAATCCAGACTACAG	182	60
	R: GCGTTGTGCGGAGGAGAATCAG		
S6K	F: ATAATCGTGCTGTGGACTGGTG	155	60
	R: TCTGGCTTCTTGTGTGAGGTAGG		
MuRF1	F: TCAACATCTACTGCGTCACCTG	128	57
	R: GCTATTCAACTCGCTCTTCTGG		
MSTN	F: GCACTGGTATTTGGCAGAGTATT	142	60
	R: TCACCTGGTCCTGGGAAAGT		
ACVR2	F: CCGATTTTGGTCTAGCTGTACG	143	60
	R: GTCTATTCTCAGGAAGGCGTCTC		
β-actin	F: GCTATGTCGCCCTGGATTTC	168	60
	R: CACAGGACTCCATACCCAAGAA		
GAPDH	F: AAGGCTGAGAATGGGAAAC	254	60
	R: TTCAGGGACTTGTCATACTTC		

### Western blotting

Cellular protein from different treatment myoblasts were exacted using RIPA buffer (Beyotime), then separated on a SDS–10% PAGE gel at 90 v for 1.5 h and transferred onto PVDF membranes (Beyotime) at 70 mA for 30 min. Membranes were incubated in blocking buffer (Beyotime) at 37°C for 3 h. Then membranes were probed with primary antibodies at 4°C for 16 h, and then washed four times with TBS/Tween 20 for 15 min each. Membranes were then incubated with secondary antibodies at 37°C for 2 h, washed four times with TBS/Tween 20 for 15 min each, two times with TBS for 5 min each and detected using a DAB HRP (horseradish peroxidase) colour development kit (Beyotime). Finally, antibody binding was detected using a Gel Imaging System (Bio-Rad). The primary antibodies and their information are listed as follows: anti-Akt, anti-phospho-Akt (Thr^308^), anti-mTOR, anti-S6K, anti-phospho-S6K (Ser^417^) and anti-tubulin are rabbit monoclonal antibodies (both diluted 1:1000, Beijing biosynthesis biotechnology Co., Ltd.). Anti-phospho-mTOR (serine 2448), anti-FoxO1, anti-phospho-FoxO1(Ser^256^) is a rabbit monoclonal antibody (diluted 1:1000, Cell Signaling Technology). Additionally, anti-tubulin (Beijing biosynthesis biotechnology Co., Ltd.) was used as a control reference. The secondary antibodies used were HRP-conjugated goat anti-rabbit IgG, which was both purchased from a biocompany (Beijing Biosynthesis Biotechnology Co. Ltd.).

### Statistical analysis

The relative gene expression levels were subjected to an ANOVA, and the means were compared for significance using Tukey's test. The relative expression of the proteins were calculated using the Image Lab software based on the protein bar on the membranes. All results are expressed as the mean±S.E. and analysed statistically. The ANOVA and *t* test were performed using SAS (SAS Institute, Cary, NC, USA). A *P*<0.05 was considered statistically significant.

## RESULTS

### Effects of FST transfection on duck myoblast phenotypes and PI3K/Akt/mTOR signalling pathway gene expression

**Figure 1:**
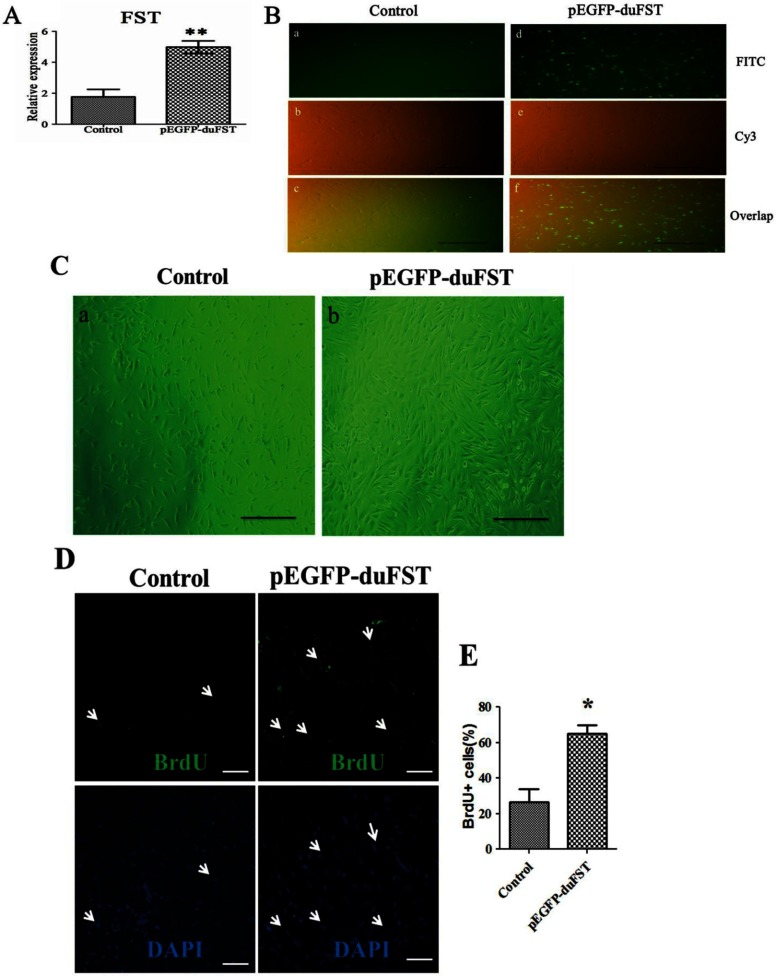
Effects of FST transfection on duck myoblast phenotypes (**A**) Relative mRNA expression of FST in duck myoblasts, the data were analysed by ANOVA and Tukey's test, the label ‘**’ indicates a significant difference (*P*<0.01). (**B**) the transfection efficiency observed with a fluorescence microscope (×100). Notes: (a), (b) and (c) are control group; (d), (e) and (f) are pEGFP-duFST-transfected group; (a) and (d) show the GFP (green fluorescent protein) in the FITC channel, (b) and (e) show the shape of the myoblasts in the Cy3 channel, and (c) and (f) show the overlap. (**C**) Representative images of myoblasts (observed by Carl Zeiss Shanghai Co., Ltd.). The pEGFP-duFST-transfected groups contained more myoblasts than the control group after 24 h transfection (×100). Notes: (a) and (b) indicate images of control and transfection group, respectively. (**D**) BrdU-labelled nuclei influenced by transfected pEGFP-duFST; all of the nuclei are labelled blue by DAPI, but only the proliferating nuclei are labelled green by monoclonal anti-BrdU (×200), the arrows shows the proliferation cells. (**E**) The number of BrdU–FITC-labelled nuclei per 100 DAPI-labelled nuclei.

As shown in Figures 1(A) and 1(B), pEGFP-duFST was successfully transfected into duck myoblasts. After the cells were transfected for 24 h, the number of myoblasts in the pEGFP-duFST group was significantly increased compared with the control group (Figure 1C). The BrdU assay was employed to analyse the proliferation states of the myoblasts, and the results showed that the percentage of BrdU-labelled nuclei was higher in the pEGFP-duFST group (Figures 1D–1E, *P*<0.05), indicating a positive role of FST on duck myoblast proliferation. In transient transfection assay, our previous research showed that pEGFP-N1 alone did not have significant effects on the myoblast proliferation, the myoblast vitality and the mRNA expression of FST [20]. Thus, we can exclude the potential influences of EGFP on the endpoint analysis.

**Figure 2:**
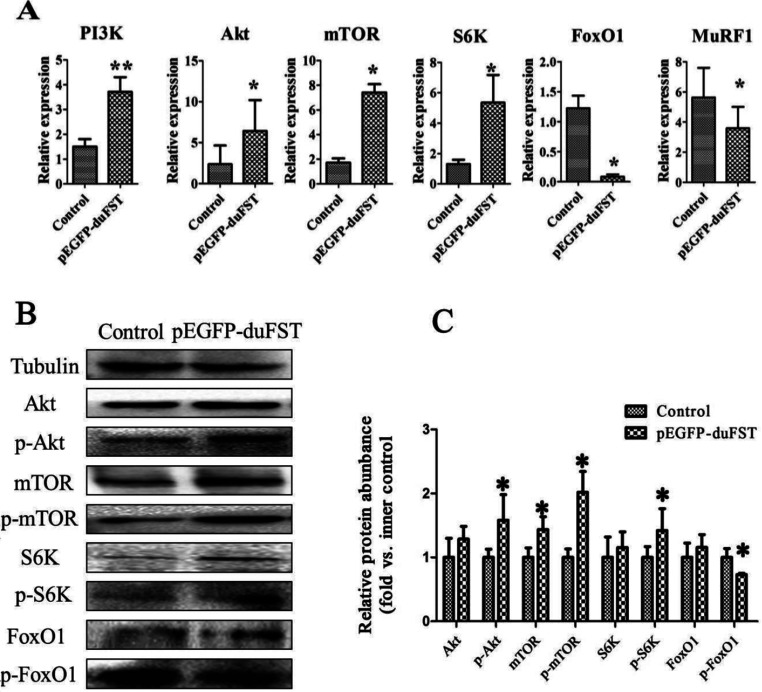
Effects of FST transfection on PI3K/Akt/mTOR pathway gene expression (**A**) Relative mRNA expression levels of PI3K, Akt, mTOR, S6K, FoxO1 and MuRF1 in transfected cells compared with control cells at 24 h, Duck β-actin and GAPDH were used as the internal controls. The data were analysed by ANOVA and Tukey's test. The label ‘*’ indicates a significant difference (*P*<0.05), and ‘**’ indicates a significant difference (*P*<0.01). The results are presented as the mean±S.E.M. (n=3). (**B**) and (**C**) The expression of Akt, phospho-Akt (Thr^308^) (p-Akt), mTOR, phospho-mTOR (serine 2448) (p-mTOR), S6K, phospho-S6K (Ser^417^) (p-S6K), FoxO1, phosphor-FoxO1 (Ser^256^) (p-FoxO1) proteins were detected by Western blot after transfection for 24 h. Each treatment and each sample were repeated in triplicate, the expression of eight proteins were normalized to the equal a value of 1 in control, *represents a significant difference (*P*<0.05).

We then determined the mRNA and the protein expression of PI3K signalling components to illustrate the possible mechanisms involved in myoblast proliferation in response to FST. We found that the overexpression of FST increased the PI3K mRNA expression levels by approximately 2.5-fold (*P*<0.01) and increased the Akt expression levels by approximately 2.7-fold (*P*<0.05). mTOR and S6K are another two molecules downstream of PI3K, and the results revealed that overexpression of FST increased the mRNA expression levels of mTOR and S6K by approximately 4.4- and 4.2-fold, respectively (*P*<0.05). On the contrary, the expression of FoxO1 and MuRF1 was inhibited by FST, and their expression levels were down-regulated by 0.94- and 0.37-fold, respectively (*P*<0.05) (Figure 2A).

We also found that overexpression of FST increased the protein expression of phospho-Akt (Thr^308^), mTOR, phospho-mTOR (serine 2448) and phospho-S6K (Ser^417^) significantly, inhibited the protein expression of phospho-FoxO1 (Ser^256^) significantly (Figures 2B and 2C), but have no significantly influence on the protein expression of Akt, S6K and FoxO1.

### Effects of LY294002 and duck FST overexpression on myoblast proliferation and PI3K/Akt/mTOR signalling

**Figure 3:**
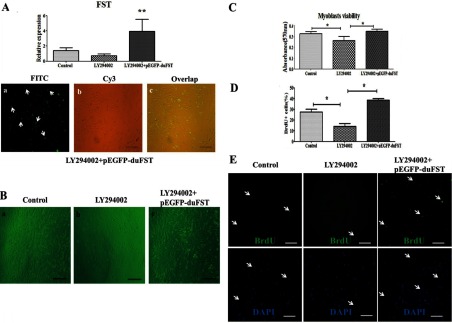
Effects of LY294002 and duck FST overexpression on myoblast proliferation (**A**) The relative mRNA expression of FST; including the control group, treatment with LY294002 for 24 h (designated as LY294002 group), transfected with pEGFP-duFST for 24 h after 24 h LY294002 treatment (designated as LY294002+pEGFP-duFST group), the data were analysed by ANOVA and Tukey's test, The label ‘**’ indicates a significant difference (*P*<0.01). The transfection efficiency of LY294002+pEGFP-duFST group was observed using a fluorescence microscope (×100). (**B**) Representative images of myoblasts for different treatments groups, (a) control group, (b) LY294002 group, (c) LY294002+pEGFP-duFST group (×100). (**C**) Proliferation myoblasts was measured by MTT assay, data are presented as the mean±S.E., n=5 wells,*(*P*<0.05). (**D**) The number of BrdU–FITC-labelled nuclei per 100 DAPI-labelled nuclei. (**E**) BrdU-labelled nuclei for different treatments group; all of the nuclei are labelled blue by DAPI, but only the proliferating nuclei are labelled green by monoclonal anti-BrdU (×200), the arrows show the proliferation cells.

Figure 3A illustrates that pEGFP-duFST was transfected into the duck myoblasts efficiently. These cells were then treated with LY294002 for 24 h. Figure 3(B) shows that the density of myoblasts in the LY294002 group was lower than the control group and the LY294002+pEGFP-duFST group. The results of the MTT assay suggest that the cell viability in the LY294002+pEGFP-duFST group was similar to the control. Both were significantly higher compared with the LY294002 group (Figure 3C; *P*<0.05) The BrdU assay illustrated a similar tendency in the proliferation rate of myoblasts among the groups, similar to the results obtained using MTT (Figures 3D–3E, *P*<0.05). All of these results suggest that duck FST overexpression rescues the inhibition of myoblast proliferation caused by LY294002.

**Figure 4:**
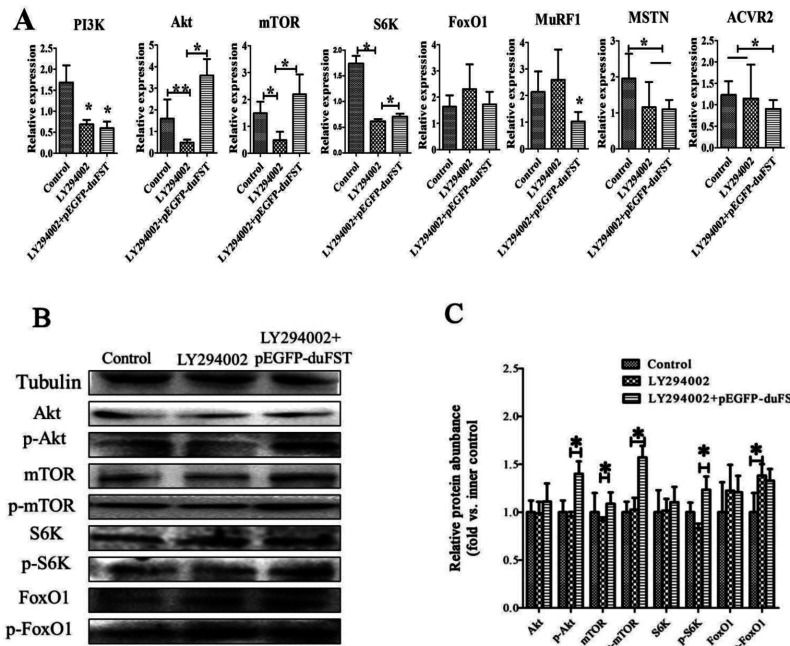
Effects of LY294002 and duck FST overexpression on PI3K/Akt/mTOR signalling (**A**) The relative mRNA expressions of PI3K, Akt, mTOR, FoxO1, MuRF1, MSTN and ACVR2 were detected by qRT-PCR and included different groups: control group, LY294002 group and LY294002 +pEGFP-duFST group. Duck β-actin and GAPDH were used as the internal controls; the data were analysed by ANOVA and Tukey's test. The label ‘*’ indicate a significant difference (*P*<0.05), and ‘**’ indicate a significant difference (*P*<0.01). The results are presented as the mean±S.E.M. (n=3). (**B**, **C**) The expression of Akt, phospho-Akt (Thr^308^) (p-Akt), mTOR, phospho-mTOR(serine 2448) (p-mTOR), S6K, phospho-S6K (Ser^417^) (p-S6K), FoxO1, phosphor-FoxO1 (Ser^256^) (p-FoxO1) proteins were detected by Western blot and included different groups: control group, LY294002 group and LY294002 +pEGFP-duFST group. Tubulin was used as the internal group. Each treatment and each sample were repeated in triplicate. The expression of eight proteins were normalized to the equal a value of 1 in control,*represents a significant difference (*P*<0.05).

The mRNA expression of PI3K was significantly down-regulated after adding LY294002 to the medium (*P*<0.05). However, the mRNA expression of PI3K was not significantly altered in the LY294002+pEGFP-duFST group. The mRNA expression of Akt (*P*<0.01), mTOR (*P*<0.05) and S6K (*P*<0.05) was markedly down-regulated in the presence of LY294002 alone and significantly up-regulated in the FST+LY294002 group (*P*<0.05). Adding LY294002 alone had no obvious effect on the mRNA expression of FoxO1, MuRF1 and ACVR2 and had an obvious inhibitory effect on the mRNA expression of MSTN (*P*<0.05). When we transfected FST in the LY294002-treated cells, the mRNA expression of FoxO1, MuRF1 (*P*<0.05), ACVR2 (*P*<0.05) and MSTN (*P*<0.05) decreased (Figure 4A).

We also found that the protein expression of phospho-Akt (Thr^308^), mTOR, phospho-S6K (Ser^417^) were down-regulated, FoxO1 and phospho-FoxO1(Ser^256^) (*P*<0.05) was up-regulated after adding LY294002 to the medium. However, when we transfected FST in the LY294002-treated cells, the protein expression of phospho-Akt (Thr^308^) (*P*<0.05), phospho-mTOR (serine 2448) (*P*<0.05), mTOR (*P*<0.05) and phospho-S6K (Ser^417^) (*P*<0.05) were significantly up-regulated significantly, the phospho-FoxO1(Ser^256^) was down-regulated (Figures 4B and 4C).

### Effects of rapamycin and duck FST overexpression on myoblast proliferation and PI3K/Akt/mTOR signalling

**Figure 5:**
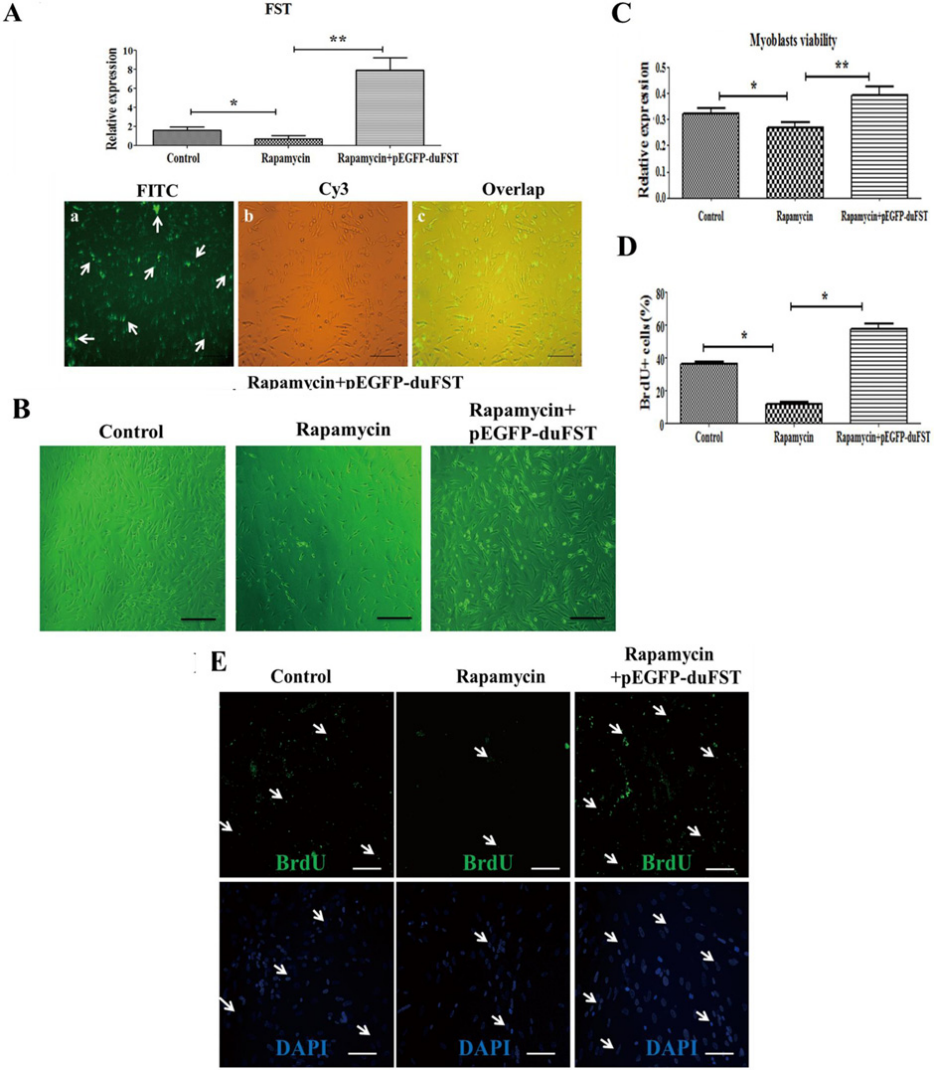
Effects of rapamycin and duck FST overexpression on myoblast proliferation (**A)** The relative mRNA expression of FST, including control group, treatment with rapamycin for 24 h (designated as rapamycin group), transfected with pEGFP-duFST for 24 h after 24 h rapamycin treatment (designated as rapamycin+pEGFP-duFST group), the data were analysed by ANOVA and Tukey's test. The label ‘*’ indicates a significant difference (*P*<0.05), and ‘**’ indicates a significant difference (*P*<0.01). The transfection efficiency of rapamycin+pEGFP-duFST was observed using a fluorescence microscope (×100). (**B)** Representative images of myoblasts for three different treatment groups (×100). (**C)** Proliferation myoblasts was measured using an MTT assay; data are presented as the mean±S.E., n=5 wells, *(*P*<0.05), **(*P*<0.01). (**D**) The number of BrdU–FITC-labelled nuclei per 100 DAPI-labelled nuclei. (**E**) BrdU-labelled nuclei for different treatment group; all of the nuclei are labelled blue by DAPI, but only the proliferating nuclei are labelled green by monoclonal anti-BrdU (×200), the arrows shows the proliferation cells.

As shown in Figure 5A, pEGFP-duFST was efficiently transfected into duck myoblasts, which were treated with rapamycin for 24 h. Figure 5(B) showed that the density of myoblasts in the rapamycin group was lower than both the control and the rapamycin+pEGFP-duFST groups. The MTT assay suggests that myoblast viability in the rapamycin group is decreased compared with the control group (*P*<0.05). Furthermore, the cell viability in both the rapamycin and control groups was significantly lower compared with the rapamycin+pEGFP-duFST group (*P*<0.01) (Figure 5C). The BrdU assay showed a similar tendency in the proliferation rate of myoblasts among the groups, similar to the results revealed by the MTT assay (Figures 5D and 5E; *P*<0.05). All of the results suggest a clear effect of duck FST overexpression in alleviating the inhibitory effects on the myoblast proliferation caused by the rapamycin.

**Figure 6:**
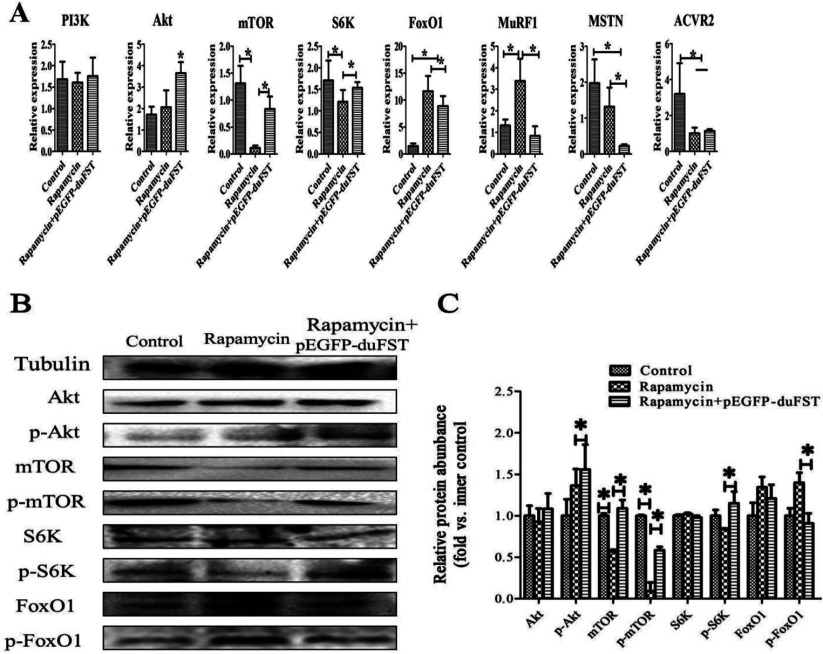
Effects of rapamycin and duck FST overexpression on PI3K/Akt/mTOR signalling (**A**) The relative mRNA expressions of PI3K, Akt, mTOR, S6k, FoxO1, MuRF1, MSTN and ACVR2 were detected by qRT-PCR and included different groups: control group, rapamycin group and rapamycin+pEGFP-duFST group. The data were analysed using ANOVA and Tukey's test. The label ‘*’ indicates a significant difference (*P*<0.05). The results are presented as the mean±SEM (n=3). (**B**, **C**) The expression of Akt, phospho-Akt (Thr^308^) (p-Akt), mTOR, phospho-mTOR(serine 2448) (p-mTOR), S6K, phospho-S6K (Ser^417^) (p-S6K), FoxO1, phosphor-FoxO1 (Ser^256^) (p-FoxO1) proteins were detected by Western blot and included different groups: control group, rapamycin group and rapamycin+pEGFP-duFST group. Tubulin was used as the internal group. Each treatment and each sample were repeated in triplicate, the expression of eight proteins were normalized to the equal a value of 1 in control, *represents a significant difference (*P*<0.05).

We then evaluated the effects of blocking mTOR with rapamycin and FST-transfection on gene expression in PI3K/Akt/mTOR signalling. Our data showed that the mRNA and the protein expression levels of mTOR and phospho-mTOR (serine 2448) were significantly down-regulated after adding rapamycin alone to the medium (*P*<0.05). However, both the mRNA and the protein expression level of mTOR, phospho-mTOR (serine 2448) were significantly up-regulated in the rapamycin+pEGFP-duFST group (*P*<0.05). PI3K and Akt are two upstream regulatory molecules of mTOR, and their expression was not significantly changed by the addition of rapamycin. In contrast, the mRNA and the phosphor-protein expression of Akt was markedly up-regulated in the rapamycin+pEGFP-duFST group (*P*<0.05). S6K, a target molecule of mTOR, the mRNA and phospho-protein expression of S6K were significantly down-regulated by the addition of rapamycin alone (*P*<0.05), but the mRNA and the phosphor-protein expression of S6K were significantly up-regulated with the FST vector and rapamycin (*P*<0.05). Other genes, including FoxO1 and MuRF1, there mRNA expression were remarkably up-regulated by adding rapamycin alone (*P*<0.05). However, their mRNA expression decreased in the rapamycin-treated and FST-transfected cells (*P*<0.05), especially the phosphor-protein expression of FoxO1 was significantly down-regulated with the FST vector and rapamycin (*P*<0.05). The mRNA expression of two main members of MSTN signalling, i.e. MSTN and ACVR2, were remarkably down-regulated after the addition of rapamycin alone (*P*<0.05). Furthermore, the expression of MSTN decreased in the rapamycin-treated and FST-transfected cells (*P*<0.05), but the expression of ACVR2 remained relatively unchanged (Figures 6A–6C).

## DISCUSSION

The proliferation of myoblasts is one of the main inducers of myogenesis during the embryogenesis period [21], and many regulators of this process were identified as having a function in myoblast proliferation [22]. Among them, FST is known to be one of the most important, and plenty of studies have highlighted its functions in promoting the proliferation and differentiation of myoblasts [5,23]. However, there is little information in birds regarding this process. We used a duck model to investigate the functions of FST in birds, and our results were consistent with the previous research in mammals, demonstrating a similar role of FST in regulating myoblast proliferation (Figure 1).

The PI3K/Akt/mTOR signalling pathway is an essential survival mechanism in a number of cell types. This pathway is widely implicated in regulating cell proliferation, such as human ovarian cancer cells and prostate cells [24–26]. FST is an important extracellular protein, and it has been shown to regulate the expression of Akt/mTOR signalling through IGF-1R, which resulted in phenotypic changes in skeletal muscle [6,27]. Considering that some studies have reported the function of FST in cell proliferation, we speculated that PI3K/Akt/mTOR signalling may be the main pathway that mediates signals from FST in myoblast proliferation. The results in the present study showed that the overexpression of FST caused significant changes. FST up-regulated the mRNA and protein expression of PI3K/Akt/mTOR/S6K signalling and down-regulated the mRNA and protein expression of FoxO1 (Figure 2), which are all important members or target genes of PI3K/Akt/mTOR signalling. Our data demonstrated that PI3K/Akt/mTOR signalling participates in the signal transduction process by mediating FST-induced phenotypic changes in myoblast proliferation.

To further confirm our speculation, LY294002 (P13K-specific inhibitor) was used to inhibit PI3K [28], which is an upstream molecule in PI3K/Akt/mTOR signalling. PI3K is a key upstream regulator of Akt [25]. It plays an important role in promoting muscle protein synthesis and is considered to be one of the essential factors involved in muscle cell proliferation [29,30]. Our results showed that inhibiting PI3K with LY294002 in duck myoblasts led to a reduction in their capability for proliferation. Treatment with LY294002 also significantly reduced the expression of PI3K, Akt, mTOR, S6K, MSTN, ACVR2 and increased the expression of FoxO1 and MuRF1. These results demonstrate that PI3K/Akt/mTOR signalling participates in a process that affects myoblast proliferation, which is similar to the research from Goncharova et al. [30], who showed that PI3K signalling is required for the proliferation of human pulmonary vascular smooth muscle cells. More importantly, we found that the overexpression of FST did not increase the expression of PI3K (inhibited by LY294002), which indicated that FST rescued the inhibition in duck myoblasts, and this rescue did not occur through altering the transcription levels of PI3K. However, FST may rescue the downstream targets genes of PI3K in myoblasts, thereby promoting myoblast proliferation. Some research showed that FST could promote skeletal muscle hypertrophy through up-regulate Smad3, Akt and mTOR in mice [31]. Therefore when PI3K/Akt/mTOR signalling was inhibited by PI3K inhibitor, FST overexpression may rescue the inhibition through Smad3, Akt and mTOR in duck myoblasts.

Next, rapamycin (mTOR-specific inhibitor) was used to block mTOR [32], which is a downstream molecule in PI3K/Akt/mTOR signalling [33]. Rapamycin has the ability to inhibit the proliferation of many cell lines [34,35]. Additionally, it was reported that rapamycin can inhibit the induction process of muscle hypertrophy [36], suggesting that mTOR plays an essential role in regulating muscle development. Our results showed that inhibiting mTOR with rapamycin in duck myoblasts led to a reduction in their capability for proliferation. Rapamycin can significantly reduce the expression of mTOR, S6K, MSTN, ACVR2 and increase the expression of FoxO1, MuRF1, without any influence on upstream regulators, including PI3K and Akt. These results indicate that in duck myoblasts, rapamycin could modulate the level of mTOR expression. This finding is consistent with previous researches, which demonstrated that rapamycin could inhibit the protein expression of mTOR and phospho-mTOR (serine 2448) in human primary NPC [37] and in rat ovary [38]. Blocking mTOR with rapamycin may further down-regulate the expression of relative genes such as S6K. This research is consistent with the research from Ohanna et al.[12], which also demonstrated that the addition of rapamycin to C2C12 medium can suppress the downstream genes of mTOR. However, rapamycin cannot influence the upstream regulators of mTOR, which is in contrast to the research from Wan et al. [39], who showed that rapamycin can activate Akt signalling through an IGF-1R-dependent mechanism in human rhabdomyosarcoma cell lines. Most importantly, we found that the overexpression of FST may rescue downstream target genes of PI3K in myoblasts, thereby promoting myoblast proliferation. To be specific, the mRNA and the phospho-protein expression of Akt, mTOR and S6K were significantly increased in the rapamycin+pEGFP-duFST group. The mechanism may be that FST could up-regulate Akt and its downstream effectors through other pathways, such as Smad3 signalling, to rescue the myoblast proliferation that was decreased by an mTOR-specific inhibitor [31]. Taken together, these results demonstrate that PI3K/Akt/mTOR signalling participates in the signal transduction process of FST-induced phenotypic changes in myoblast proliferation.

In conclusion, we found that the overexpression of duck FST could promote myoblast proliferation and rescue the inhibition (treatment with LY294002 and rapamycin) of myoblast proliferation. Our study demonstrated that duck FST could promote myoblast proliferation, dependent on the PI3K/Akt/mTOR signalling. These novel findings will advance our understanding of the regulatory mechanisms controlling duck FST in skeletal muscle development.
